# Long-term memory, synaptic plasticity and dopamine in rodent medial prefrontal cortex: Role in executive functions

**DOI:** 10.3389/fnbeh.2022.1068271

**Published:** 2023-01-11

**Authors:** Denis Sheynikhovich, Satoru Otani, Jing Bai, Angelo Arleo

**Affiliations:** ^1^Sorbonne Université, INSERM, CNRS, Institut de la Vision, Paris, France; ^2^Institute of Psychiatry and Neuroscience of Paris, INSERM U1266, Paris, France

**Keywords:** synaptic plasticity, long-term memory, dopamine, prefrontal cortex, neuromodulation, executive functions, behavioral flexibility, computational models

## Abstract

Mnemonic functions, supporting rodent behavior in complex tasks, include both long-term and (short-term) working memory components. While working memory is thought to rely on persistent activity states in an active neural network, long-term memory and synaptic plasticity contribute to the formation of the underlying synaptic structure, determining the range of possible states. Whereas, the implication of working memory in executive functions, mediated by the prefrontal cortex (PFC) in primates and rodents, has been extensively studied, the contribution of long-term memory component to these tasks received little attention. This review summarizes available experimental data and theoretical work concerning cellular mechanisms of synaptic plasticity in the medial region of rodent PFC and the link between plasticity, memory and behavior in PFC-dependent tasks. A special attention is devoted to unique properties of dopaminergic modulation of prefrontal synaptic plasticity and its contribution to executive functions.

## 1. Introduction

Any complex purposeful behavior relies on both online control processes and a long-term representation of related previous experience, including its spatio-temporal context and associated goals, strategies or rules. Mnemonic functions supporting these two aspects of executive control can be separated on the basis of relevant time scales and underlying neural mechanisms (Atkinson and Shiffrin, 1971; Cowan, 2008). Online control processes, including, e.g., decision making, conflict monitoring, and active maintenance of goals or rules, rely on working memory, lasting from seconds to minutes and thought to be supported by reverberatory population activity, bistability in single cells or short-term synaptic changes (Durstewitz et al., 2000; Mongillo et al., 2008). In contrast, long-term storage of contextual representations is implemented by synaptic changes that last from tens of minutes to years and involve increases in receptor density, protein synthesis and structural changes at the synaptic site (Davis and Squire, 1984; Bliss and Collingridge, 1993; Yuste and Bonhoeffer, 2001).

PFC is a central structure mediating executive functions (Gilbert and Burgess, 2008) and neuronal mechanisms supporting these functions can also be studied from the processing or representational perspectives (Wood and Grafman, 2003). The discovery of cells with “memory fields” in the primate dorsolateral PFC (Fuster and Alexander, 1971; Goldman-Rakic, 1995), which fired with maximal rates when a stimulus presented at a specific location of the visual field was held in memory during a delay, stimulated an extensive study of the role of working memory in executive functions over the last few decades (Arnsten, 2013). This research led to a considerable progress in our understanding of neuronal mechanisms underlying online control of behavior and their possible implication in related mental disorders (Durstewitz et al., 2000; O'Reilly and Frank, 2006; Rolls et al., 2008). In contrast, the involvement of prefrontal *long-term* memory in executive functions received relatively little attention. While a general implication of PFC in memory has been demonstrated in different species in various experimental paradigms, in what ways the prefrontal long-term plasticity can be directly involved in the control of behavior is a matter of debate (Blumenfeld and Ranganath, 2007; Euston et al., 2012). The main questions that can be asked in relation to this involvement are:

- What aspects of executive function rely on prefrontal long-term memory?
- What molecular and neuronal mechanisms support such a long-term memory storage?

While the main issue at stake concerns the role of intrinsic prefrontal memory storage mechanisms in human executive functions, a direct experimental study of these questions in primates is difficult. This difficulty stems from the fact that even when PFC neurons are shown to encode task-related information using single cell recordings or neuroimaging methods, it is hard to prove that such information is stored in the PFC and not in other areas with which it is connected. A case in point is the delay-period activity of primate prefrontal cells mentioned above: even though these cells have been studied for several decades now, it is not clear whether their activities are directly associated with remembered stimuli or represent top-down control signals to other (e.g., sensory) cortices that retain stimulus-specific information in memory (Lara and Wallis, 2015). It appears then that rodent models can be of help, since they are readily amenable to electrophysiological, pharmacological and genetic methods in order to test both the memory contents and its storage site. One difficulty of using rodents to understand primate executive functions is that no area in the rodent brain is anatomically homologous to the primate “granular” PFC (including the dorsolateral region), thought to support complex behavioral control (Wise, 2008; Carlén, 2017). However, a large body of research shows that rodent medial PFC (mPFC) mediates many functions attributed to the dorsolateral PFC in primates, suggesting that understanding neuronal mechanisms of rodent executive function, including its long-term memory component, can provide valuable insights into its organization in primates (Brown and Bowman, 2002; Dalley et al., 2004; Chudasama, 2011). In particular, synaptic plasticity research over the last decades provided a considerable amount of data describing in which behavioral tasks and under what experimental conditions prefrontal synapses undergo long-term changes. In parallel, theoretical models of long-term plasticity involvement in behavioral control, in PFC as well as in other areas, and at various levels of physiological detail, suggested ways in which plasticity can be linked with behavior. By unifying available experimental and theoretical evidence, this review attempts to address the above questions from the synaptic plasticity perspective.

The first part of this review describes pharmacological, electrophysiological and behavioral data supporting a direct link between long-term memory and synaptic plasticity in rodents, in experimental paradigms that were shown to depend on the integrity of mPFC. The second part presents a detailed account of prefrontal synaptic plasticity mechanisms, *in vivo* and *in vitro*, in mice and rats, demonstrating that neurons in this structure readily exhibit plastic changes in a variety of experimental protocols. A well-known property of neural functioning in mPFC is that it is strongly modulated by the neuromodulator dopamine, to the extent that it is hardly possible to study prefrontal synaptic plasticity and memory without taking into account dopamine involvement (Jay, 2003; Otani, 2004). The third part of the review is therefore devoted to the properties of dopaminergic modulation of prefrontal long-term plasticity. The fourth part compares theoretical models of synaptic plasticity modulated by dopamine in the mPFC, the striatum and the hippocampus, areas that are often co-involved with mPFC in behavioral tasks that tax executive functions (Floresco et al., 1997; Kesner and Rogers, 2004). The review is concluded by attempting to provide answers to the two questions above in the context of executive functions research in rodents.

## 2. Long-term memory in mPFC and its role in rodent behavior

In an influential review, Wood and Grafman (2003) classified existing models of PFC according to whether they adopt a processing or a representational approach to describe the functional role of this structure in cognition. The processing approach attempts to characterize algorithmic procedures (e.g., selection of goals and rules to achieve them) governing the active control of behavior. These procedures are usually considered to be independent of the nature of the stimuli they operate with, so that knowing *what* is stored in prefrontal long-term memory is not essential for understanding PFC function from this point of view. In contrast, the representational approach focuses on the type information stored by the PFC, as it is this information that distinguishes it from other cortical structures and defines its functional significance (as, e.g., memory about objects and faces is an essential function of the inferior temporal cortex). The authors argue that the representational approach is more general (since the characterization of the kind of processes that PFC stores fits it), more in line with evolutionary history of PFC and its anatomical properties (see also Wise, 2008) and potentially more fruitful in generating experimentally testable predictions. This work is one of the first to highlight long-term memory as an important issue in prefrontal research. Another line of evidence supporting the important role of PFC, and in particular of its medial region, in long-term memory comes from studies of memory consolidation (see Frankland and Bontempi, 2005, for review). Based on the combined imaging and inactivation methods in an animal model of retrograde amnesia, it is proposed that mPFC plays an essential integrative role in storage and recall of remote memories, complementing the hippocampus that is thought to store primarily recent ones. More recently, Euston et al. (2012) reviewed evidence of mPFC involvement in the processing of both recent and remote memories and proposed that its primary role is to learn associations between the context (including location and events) and adaptive responses.

The direct involvement of mPFC in the storage of recent and remote memories is supported by evidence showing that manipulation of specific molecular targets disrupts memory in this and nearby cortices. In particular, blockade of muscarinic and N-Methyl-D-aspartate (NMDA) receptors in the prelimbic mPFC immediately after learning an odor-reward association induced severe memory impairment 1 or 2 days later (Tronel and Sara, 2003; Carballo-Márquez et al., 2007). Protein synthesis blockade with anisomycin (as well as NMDA receptor disruption) in the same region blocked consolidation of object recognition memory 1 day after learning (Akirav and Maroun, 2006). In the nearby orbitofrontal cortex, disruption of the synapic plasticity cascade mediated by mitogen-activated protein kinases (MAPK) and extracellular signal-regulated kinases (ERK) blocked remote olfactory memory and abolished the late development of cortical structural plasticity (Lesburguères et al., 2011), whereas in the frontal association cortex learning and extinction caused elimination and formation of dendritic spines at the same dendritic branches (Lai et al., 2012). Cortical storage of remote taste memory has been associated with protein kinase M zeta (Shema et al., 2007), whereas remote fear memory was blocked in α-CaMKII^+\−^ mice, in which cortical, but not hippocampal, synaptic plasticity is impaired (Frankland et al., 2001, 2004).

**Figure 1:**
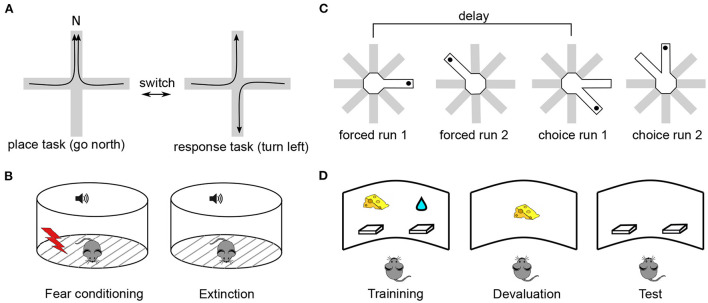
Typical experimental paradigms in which performance depends on synaptic plasticity in the mPFC. **(A)** Strategy selection. Rodents are trained to switch between place and response tasks, following a switch of reward contingency (Rich and Shapiro, 2007). In the place task, animals are rewarded if they reach the same arm (e.g., N) starting from any other arm. In the response task, reward is given if animals perform the same body turn at the intersection point. Importantly, the contingency switch is not signalled by any external cue, the animals have to infer it *via* trial-and-error. In a *set-shifting* version of the task (Birrell and Brown, 2000), animals switch between decisions based on different types of cue (e.g., odor vs. texture), instead of spatial strategy. **(B)** Fear extinction. During a conditioning period, animals associate a neutral cue, e.g., a sound tone, with a footshock, so that they acquire a freezing response to the cue (Herry and Garcia, 2002). During an extinction period the cue is repeatedly presented without the footshock, so that animals “learn to forget” the freezing response. In a *contextual* fear conditioning, the fear response is associated with environmental features (e.g., its visual appearance) rather than a discrete cue (Bouton et al., 2011). **(C)** Delay-period working memory. In an 8-arm maze, animals retrieve a reward (black dot) from a single open arm (forced choice 1) (Touzani et al., 2007). After a delay, the same arm is open together with an adjacent one and the animal has to choose the non-visited arm to obtain the reward (choice run 1). In a more complicated version of the task, a second forced run is inserted during the delay so that the animal has to keep in memory the two arms and use this information after the corresponding delay. **(D)** Decision making. During a training phase, animals learn to press on either of the two levers to obtain distinct rewards associated with them (e.g., a food pellet or sucrose solution, Corbit and Balleine, 2003). During the following devaluation phase, the animals are fed to satiety on one of the two reward types. During test, no reward is given and the preponderance of the animals to press on either lever is measured. Goal-directed behavior corresponds to a less frequent use of the lever associated with the devaluated reward. In a more complex version of the task, one employs *contingency degradation* instead of reward devaluation. In that case, the animal is given a particular reward irrespective of whether it presses on the associated lever or not, leading to a less frequent use of this lever in normal animals.

The above data strongly suggest that mPFC, similarly to nearby cortical areas, directly participates in associative long-term memory, at least in rodents. However, in which way this memory supports flexible control of behavior is less clear. Rodent studies of *behavioral flexibility*, a term that is often used to describe the kind of cognitive functions referred to as “executive” in human and primate literature, can be separated into four main classes according to whether they test strategy selection (or set-shifting), extinction learning, delay-period working memory or decision making (Figure 1). In all these paradigms, (i) Successful performance depends on mPFC integrity and requires task acquisition across hours/days; (ii) Task learning is associated with neuronal activity changes and synaptic plasticity within mPFC; and (iii) Experimental manipulations of synaptic plasticity during or after learning perturbs behavioral performance. These properties make it possible to study which aspects of flexible behavior rely on long-term synaptic plasticity within mPFC.

In the spatial *strategy-selection* paradigm or its non-spatial version referred to as *set-shifting*, animals are trained to flexibly switch from one behavioral strategy to another following a sudden change in the stimulus-reward contingency (de Bruin et al., 1994; Ragozzino et al., 1999; Birrell and Brown, 2000; Floresco et al., 2006; Rich and Shapiro, 2007). These paradigms were developed specifically as adaptations of cognitive flexibility studies in primates (such as the Wisconsin Card Sorting Task and its monkey analogs, Berg, 1948; Mansouri et al., 2006) to rodents (Ragozzino et al., 1999; Bissonette et al., 2013). In the spatial version of the task (Figure 1A), animals are initially rewarded when they follow a “place-based” strategy, approaching the same unmarked allocentric location in the maze from different starting positions. Once this strategy is successfully acquired, the experimenter switches (unknown to the animal) the reward contingency so that animals are now rewarded when they follow a different, “response-based” strategy. Here, the animals are rewarded when they make decisions based on immediately perceived cues, for example, “once at the center of the maze, turn left,” or “go to the arm with a yellow landmark at the end.” While learning of the strategies themselves is not impaired by mPFC inactivation, the switching between those strategies is (Ragozzino et al., 1999; White and McDonald, 2002). In set-shifting, animals first learn to choose one of two feeding bowls to dig for food, based on a particular type of perceptual cue (e.g., the odor of the digging medium). After the contingency switch, they have to make choices based on a different type of cue, e.g., the texture of the bowl surface. mPFC lesions selectively impair shifting of the perceptual set, but not its acquisition (Birrell and Brown, 2000). In these tasks, learning is associated with neural activity changes in mPFC (Rich and Shapiro, 2009; Durstewitz et al., 2010; Singh et al., 2019) and with plasticity-related gene expression in mPFC (DeSteno and Schmauss, 2008; Burnham et al., 2010). The main learning components of these tasks are the selection of a new behavioral strategy and inhibition of a previously acquired one, following a contingency switch (Dalley et al., 2004). Different mPFC subregions are differentially involved in mediating these components, depending on the nature of the task (Kesner, 2000; Floresco et al., 2009; Bissonette et al., 2013).

*Extinction learning* can be considered as a type of behavioral flexibility, in which a previously acquired emotional response (e.g., footshock-induced freezing) to a discrete stimulus (e.g., a sound) or to a contextual cue (e.g., an environment) is learned to be inhibited (Morgan and LeDoux, 1995; Quirk and Mueller, 2008; Bouton et al., 2011; Gass et al., 2014) (Figure 1B). In primates, fear extinction studies are motivated by the general question of emotional regulation of behavior and, in particular, by the role of stress in the development of many psychiatric disorders, including post-traumatic stress disorder (Sotres-Bayon et al., 2006; Maren and Holmes, 2016). In early studies of the role of mnemonic processes in fear conditioning, synaptic changes in hippocampal-mPFC synapses were shown to occur during the acquisition of an associative task using tone-shock pairings (Doyère et al., 1993). Moreover, an increase in plasticity-related immediate-early gene expression (c-fos) in mPFC during a similar task was reported, while its suppression was found to produce learning deficits (Morrow et al., 1999). However, subsequent investigations revealed the key role of the basolateral amygdala in associating sensory and shock-related inputs (Quirk and Mueller, 2008) and a hippocampal involvement in the reinstatement of extinguished fear (Frohardt et al., 2000; Short et al., 2022), whereas mPFC was shown to mediate primarily the consolidation of extinction. More specifically, the learning to inhibit a fear response was shown to be associated with synaptic changes in mPFC, as tested by an electric stimulation of medio-dorsal thalamus or ventral hippocampus synapses to mPFC neurons (Milad and Quirk, 2002; Quirk et al., 2006; Hugues and Garcia, 2007). Moreover, behavioral extinction learning was sped up or slowed down by an electric stimulation of monosynaptic thalamus-mPFC projections (Herry and Garcia, 2002). Finally, local pharmacological block of NMDA receptors, MAPK/ERK pathway or protein synthesis prevented long-term extinction (Runyan, 2004; Santini, 2004; Hugues et al., 2006; Burgos-Robles et al., 2007; Mamiya et al., 2009) and inhibitory avoidance (Zhang et al., 2011). Potentially different mPFC subregions learn different components of fear response (i.e., expression of fear response vs. inhibition of fear response) similarly to what has been proposed for set-shifting (Quirk and Mueller, 2008).

In a *delay-period working memory* paradigm, adapted from the behavioral paradigm of the same name in primates (Arnsten, 2013), rodents retain in memory a trial-unique spatial information from a training phase. During a test phase, that starts after a delay, they are required to retrieve this information and use it to solve the task (Floresco et al., 1997; Horst and Laubach, 2009). The delay duration varies from 3 s to 30 min in different experiments in rodents, while in primate studies it is at most several seconds. While some lesion studies have shown that intact mPFC is necessary for successful performance in delay-period tasks (Horst and Laubach, 2009), others reported that mPFC is not needed for information storage during the delay and it is only required during spatial information retrieval and use for guiding prospective action (Floresco et al., 1997; Seamans et al., 1998; Gisquet-Verrier and Delatour, 2006). Neuronal activity dynamics in mPFC were shown to reflect learning during a delayed alternation task (see, e.g., Baeg et al., 2003) and in one study, optogenetic suppression of enhanced activity of pyramidal neurons in mouse mPFC impaired learning (Liu et al., 2014). It is not clear whether long-term plasticity within mPFC is involved in simple delay-period memory tasks, but in one study it was shown that a day-to-day improvement in delayed non-matching to sample task with retroactive interference, performance improvement required protein synthesis in mPFC (Touzani et al., 2007 see Figure 1C, see also Marighetto et al., 2008). In a related reference-memory experimental paradigm, early studies have shown that learning was associated with plasticity-related gene expression in hippocampal-prefrontal synapses (Davis et al., 1996; Laroche et al., 2000).

Finally, *decision making* is often tested in rodents using a reward devaluation or contingency degradation experimental paradigms (Corbit and Balleine, 2003; Killcross and Coutureau, 2003). These paradigms are based on the distinction between goal-directed and habitual behaviors, the former of which is mediated by mPFC in rodents and primates (Balleine and O'Doherty, 2010; Dolan and Dayan, 2013). During a goal-directed behavior, goal information is directly associated with actions (or chains of actions) leading to it, such that a change in the goal value or in the action-reward contingency induces changes in the corresponding behavior. In contrast, habitual behaviors prescribe action choices that are linked to the context or a cue associated with the goal, regardless of the internal motivation for the goal. Thus, if an animal solves a task in a goal-directed manner, reward devaluation (e.g., by feeding to satiety) should lead to a less probable choice of specific actions leading to it, reflecting the flexibility in the choice of actions (Figure 1D). However, if responses are habitual, rather than goal-directed, the actions leading to the now devaluated goal will be automatically repeated. Lesion studies support the implication of (prelimbic) mPFC in goal-directed behavior by showing a strong bias of lesioned animals toward habitual actions (Corbit and Balleine, 2003). Moreover, learning in decision making tasks occurs across several days (Killcross and Coutureau, 2003) and is associated with learning-related changes in response patterns of prefrontal neurons (Mulder et al., 2003). Lastly, the implication of synaptic plasticity mechanisms, intrinsic to mPFC, in goal-directed decision-making tasks was demonstrated by showing an increased expression of MAPK/ERK in mPFC following learning, as well as by the fact that local inhibition of this pathway prevented learning (Hart and Balleine, 2016). Moreover, augmentation of dendritic spine plasticity using cytoskeletal regulatory factor Rho kinase inhibitor stimulated goal-directed behavior in mice compared to control animals who expressed stimulus-response habits (Hart and Balleine, 2016; Swanson et al., 2017; Woon et al., 2020). In spatial goal-directed tasks, a long-term goal representation in mPFC neurons has been observed (Poucet et al., 2004; Hok et al., 2005), whereas inhibition of the MAPK plasticity pathway in the mPFC immediately after training impaired performance in a version of the Morris watermaze task (Leon et al., 2010).

The experimental data above support the proposal that rodent mPFC is directly involved in a behaviorally important long-term memory storage. In relation to the first question asked in the Introduction, the primary candidate options for executive long-term storage in rodents are high-level representations of behavioral strategies (Ragozzino et al., 1999; Rich and Shapiro, 2009) and rules for switching between them (Durstewitz et al., 2010; Hyman et al., 2012), necessary prerequisites of behavioral flexibility (Granon and Floresco, 2009). In spatial navigation tasks, neuronal representations of different strategies correspond to strategy-selective populations of neurons that are activated when memory systems, encoding these strategies, are engaged in the current behavioral task (White and McDonald, 2002; Arleo and Rondi-Reig, 2007; Rich and Shapiro, 2009). In fear conditioning, both the expression of learned fear response and the expression of learned extinction are stored in the distributed network of interconnected structures, including mPFC, which exerts control over other structures such as amygdala (Quirk and Mueller, 2008). The data showing that long-term memory in mPFC is required for complex working-memory tasks suggest that it can keep a memory trace of a mental strategy (Otani et al., 2002), instrumental to “working with memory” (Seamans and Yang, 2004).

Whether a contextual information, either spatial (i.e., related to a representation of surrounding environment during a particular task) or non-spatial (i.e., related to temporal or other aspects of the task), is also stored in the mPFC, may depend on the nature and complexity of the task (Euston et al., 2012; Hyman et al., 2012). In complex spatial tasks or in non-spatial tasks, mPFC has been repeatedly implicated in supporting a high-level hierarchical representation of the environment or the abstract task model (Tanji and Hoshi, 2001; Botvinick, 2008), which can be considered as a more complex form of contextual task representation. In the analysis of reward devaluation experiments, a distinction between goal-directed and habitual actions, is often considered in terms of the distinction between “model-based” and “model-free” algorithms in the reinforcement learning literature (Dayan and Niv, 2008). Thus, in addition to a representation of rules or strategy switches, it is likely that during complex tasks prefrontal memory circuits store a high-level “task model,” e.g., as a topological graph of the environment (Hasselmo, 2005; Martinet et al., 2011) or as a tree-like decision structure (Daw et al., 2005), see Section 5. This proposal is supported by experimental studies showing that mPFC is involved in the memory of spatial goals (Hok et al., 2005) and of a temporal order of spatial information in complex tasks (Kesner, 2000).

## 3. Long-term synaptic plasticity in mPFC

Assuming, based on the considerations above, that long-term memories are indeed stored in mPFC and support its role in flexible control of behavior, the second question asked in the Introduction addresses neuronal mechanisms underlying the formation of such memories, i.e., long-term depression (LTD) and long-term potentiation (LTP) (Bliss and Collingridge, 1993). As in many other cortical areas, neurons in mPFC readily exhibit long-lasting changes in synaptic strength under a variety of different experimental protocols, thus providing mechanistic support for the hypothesis of long-term memory storage in this brain structure.

In general, synaptic stimulation at low and high frequencies is expected to result in LTD and LTP, respectively, in agreement with the standard synaptic plasticity model described by the Bienenstock-Cooper-Munro (BCM) theory of cortical plasticity (Bienenstock et al., 1982; Lisman, 1989; Cooper et al., 2004). A prominent feature of this theory is the adaptive threshold between LTD and LTP, the value of which determines the direction (i.e., depression or potentiation) of plasticity at a given stimulation strength (or, alternatively, at a given level of stimulation-induced intra-synaptic calcium concentration). The LTD-LTP pattern of dependence on stimulation frequency has also been observed in rodent mPFC, although with a number of differences between rats and mice in terms of synaptic stimulation protocols, required to induce plasticity, and underlying molecular mechanisms. Studies reviewed below are summarized in Table 1, according to the species, type of plasticity and the corresponding induction protocol.

**Table 1:** Experimental studies on long-term synaptic plasticity in rodent PFC.

**Rats**					
	**Type**	**Freq. (Hz)**	**Protocol**	**Input**	**References**
*In vivo*	LTP	250	tet	HPC	Laroche et al., 1990
		50	tet	HPC	Mulder et al., 1997
		10–200	tet	Contr	Gemmell and O'Mara, 2000
		50	tet	Vis	Kim et al., 2003
		5 × 100	BS	AM	Maroun and Richter-Levin, 2003
	LTD	1 × 250	BS	HPC	Takita et al., 1999
		1 × 250	BS	HPC	Izaki et al., 2000
*In vitro*	LTP	300	tet	II-III	Huang et al., 2004
	LTD/LTP	50–100	tet	I-II	Hirsch and Crepel, 1991
		5–100	BS	I–II	Vickery et al., 1997
		50	tet	I–II	Kolomiets et al., 2009
	LTD	1 × 20	PP	II–III	Huang and Hsu, 2010
		1 × 20	PP	I–II	Caruana et al., 2011
		3	tet	I–II	Bai et al., 2014
**Mice**					
*In vivo*	LTP	250	tet	MdT	Herry and Garcia, 2002
	LTD	2	tet	MdT	Herry et al., 1999
*In vitro*	LTP	300	tet	II-III	Huang et al., 2004
		100	tet, BS	II–III	Xu et al., 2009
		100	tet, BS	II–III	Cui et al., 2011
	LTD	3	tet	II-III	Huang et al., 2004
		10	tet	II–III	Lafourcade et al., 2007

Protocols: tetanic stimulation (tet), burst stimulation (BS), paired-pulse protocol (PP). For tetanic stimulation, frequency in Hz is given; for burst stimulation and paired-pulse protocol, inter-burst frequency is indicated first and then burst frequency. In all experiments, layer V pyramidal neurons were recorded, while the stimulation site is indicated in the Input column (roman numbers refer to the cortical layer where the input stimulation was delivered). HPC, hippocampus; Contr, contralateral prelimbic cortex; Vis, visual cortex; AM, amygdala; MdT, medio-dorsal thalamus. Only one reference per laboratory is given if the same experimental protocol is used in several studies.

### 3.1. Prefrontal synaptic plasticity in the rat

***In vivo***. In anesthetized rats, a tetanic stimulation at frequencies 50–250 Hz induced LTP of projections from the hippocampus, the visual cortex, the amygdala and the contralateral cortex to the prelimbic subregion of the mPFC. In particular, in synaptic contacts from hippocampal afferents originating in the CA1 or subiculum, LTP was induced by tetanic stimulation at 250 Hz (Laroche et al., 1990; Jay et al., 1996, 1998; Mulder et al., 1997). This LTP required NMDA receptor activity and protein kinase A (PKA). As shown below, the implication of NMDA-PKA molecular cascade, also participating in the classical hippocampal LTP (Malenka and Bear, 2004), is a common feature of prefrontal LTP induced at high frequencies in rats and mice. In projections from the contralateral prelimbic cortex, LTP was induced at frequencies 10–200 Hz (Gemmell and O'Mara, 2000), and in those from the visual cortex at 50 Hz (Kim et al., 2003). In synaptic contacts from the amygdala, LTP was induced by theta burst stimulation (Maroun and Richter-Levin, 2003).

Standard low-frequency tetanus protocols have initially failed to induce LTD in the synapses from the ventral hippocampal CA1 to the prelimbic cortex (Burette et al., 1997). However, Takita et al. (1999) determined a reliable LTD-inducing low-frequency (1 Hz) burst protocol by varying the burst duration, thus supporting the LTD-LTP plasticity pattern (see also Izaki et al., 2002, 2003).

It is interesting to note here that even though Burette et al. (1997) did not observe LTD following a number of low-frequency protocols, one of them (2-pulse 5 ms bursts delivered at 1 hz) resulted in a depotentiation of a previously induced LTP. This depotentiation decreased synaptic strength to the baseline, but not below it, even after the same stimulation was repeated several times. Depotentiation was initially observed in the hippocampus (Malenka and Bear, 2004) and is considered as a possible forgetting, or extinction, mechanism (Kim et al., 2007). Even though it is largely accepted that extinction constitutes new learning rather than erasure of the old one (Bouton et al., 2011), a recent study has shown that opposite changes at the same dendritic branches in the frontal cortex are associated with fear learning and forgetting (Lai et al., 2012).

***In vitro***. Layer V pyramidal neurons in rat mPFC slices were recorded either intracellularly (Hirsch and Crepel, 1990; Vickery et al., 1997; Otani et al., 1998; Caruana et al., 2011) or extracellularly (Huang et al., 2004), while an electric stimulation was delivered by extracellular current pulses to layers I–II or layers II–III, respectively. These layers contain synapses of cortico-cortical projections (from neighboring neurons, contralateral mPFC neurons, and from other cortical areas), as well as afferent fibers from a wide variety of subcortical brain structures (including the hippocampus, Kuroda et al., 1998; Hoover and Vertes, 2007).

Similarly to the *in vivo* studies cited earlier, a high-frequency stimulation (300 Hz) induced NMDA-dependent LTP in these synapses, and pharmacological block of PKA or protein synthesis impaired this LTP (Huang et al., 2004). Lowering stimulation frequencies to 50–100 Hz resulted in high neuron-to-neuron variability in terms of the sign and amplitude of plasticity, as different neurons expressed either LTP, LTD or no change under this protocol (Hirsch and Crepel, 1990; Law-Tho et al., 1995; Vickery et al., 1997; Auclair et al., 2000). While calcium elevation was necessary for both LTD and LTP at these intermediate frequencies (Hirsch et al., 1992), a pharmacological NMDA receptor blockade masked LTP and resulted in LTD instead (Hirsch and Crepel, 1991). In a series of studies, in which tetanic stimulation at a fixed frequency of 50 Hz was used, either no change (when the stimulation included 4 tetanic trains) or NMDA-independent LTD (with 6 tetanic trains) was observed (Otani et al., 1998, 1999; Kolomiets et al., 2009). This variability of plastic changes at the intermediate stimulation frequencies can be interpreted as a consequence of proximity of synaptic stimulation (or of stimulation-induced synaptic state) to the LTD/LTP threshold. Finally, synaptic stimulation at still lower frequencies reliably resulted in LTD (Huang and Hsu, 2010; Caruana et al., 2011; Bai et al., 2014). In particular, two different forms of activity-dependent LTD were observed: one that required NMDA receptor activation, and the other that did not. The first, NMDA-dependent form of LTD, was reported when plasticity was induced by a tetanic stimulation at 3 Hz (Bai et al., 2014). This form of LTD involved phospholipase C-protein kinase C (PLC-PKC) molecular cascade and MAP kinase activity. The second, NMDA-independent form of LTD, was observed following a paired-pulse stimulation at 1 Hz (Huang and Hsu, 2010; Caruana et al., 2011), and required an activation of metabotropic glutamate receptors (mGluRs), muscarinic acetylcholine receptors and PKC.

### 3.2. Prefrontal synaptic plasticity in the mouse

In the mouse *in vivo*, a high-frequency (250 Hz) stimulation of mediodorsal thalamic afferents resulted in LTP of stimulation-evoked response approximately 30 min after the stimulation (Herry and Garcia, 2002), while a low-frequency (2 Hz) stimulation of mediodorsal thalamus afferents resulted in LTD or LTP in different mice (Herry et al., 1999; Herry and Garcia, 2002). However, when the same stimulation was combined with fear conditioning, a reliable LTD was observed (probably due to a neuromodulatory effects on plasticity, see below).

In the mouse *in vitro*, a 100–300 Hz tetanic and theta-burst stimulation delivered to mPFC layer II-III, as well as a pairing protocol for the same synapses also resulted in LTP (Huang et al., 2004; Xu et al., 2009), which was NMDA-dependent (Cui et al., 2011). At low stimulation frequencies (3 and 10 Hz), LTD was observed in the same synapses that did not require NMDA activation, but was instead mGluR-dependent and required endocannabinoid receptor activation (Huang et al., 2004; Lafourcade et al., 2007).

### 3.3. Summary of experimental results on synaptic plasticity using BCM curves

**Figure 2:**
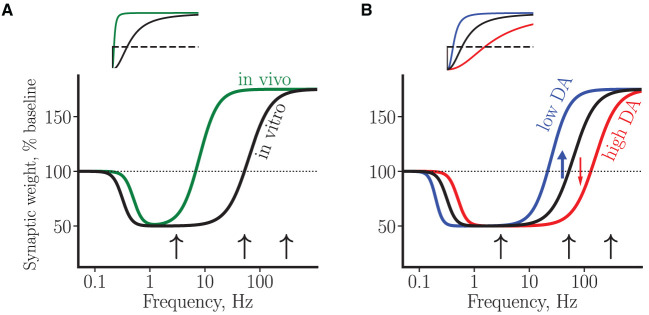
A schematic summary of synaptic plasticity experiments using BCM curves. **(A)** Plasticity curves corresponding to *in vivo* (green line) vs. *in vitro* (black line) data. The LTD/LTP threshold *in vivo* has a lower value and the LTD window is narrower. **(B)** Plasticity curves corresponding to low (blue line) and high (red line) tonic DA concentrations, for the *in vitro* condition. The black curve in **(B)** is a copy of the curve in **(A)**. The blue and red arrows illustrate D1 facilitation of LTP and D2 facilitation of LTD by low and high DA concentration, respectively, near plasticity threshold. In both plots, the vertical axis corresponds to a change in average synaptic weight of a neuron, measured by the amplitude of somatic EPSP. The horizontal axis represents stimulation frequencies on a logarithmic scale. The black arrows correspond to a stimulation at low (3 Hz), intermediate (50 Hz) and high (300 Hz) frequencies. Insets: the same curves as in the main plots, shown on a linear scale.

Within the framework of the BCM theory of synaptic plasticity mentioned earlier, the above data can be interpreted in terms of plasticity curves that schematically represent changes in synaptic efficacy as a function of presynaptic stimulation strength (or, equivalently, as a function of stimulation-induced post-synaptic depolarization or calcium elevation, Bear et al., 1987; Lisman, 1989; Kirkwood et al., 1996), see Figure 2A. Thus, a high-frequency stimulation invariably results in LTP, while a low-frequency stimulation leads to LTD, with the LTD/LTP threshold somewhere in between these two regimes. The experimentally observed neuron-to-neuron variability with respect to plasticity amplitude and direction at intermediate frequencies (i.e., in the range of 50–100 Hz *in vitro* and 3–10 Hz *in vivo*) may thus reflect proximity of stimulation-induced activation of molecular plasticity processes to the intrinsic neuronal LTD/LTP threshold. This interpretation suggests the LTD/LTP threshold *in vivo* is shifted toward lower synaptic activity levels and/or calcium concentrations. In addition, the difficulty of choosing the right protocol for LTD induction *in vivo* can be interpreted as corresponding to a narrower LTD window.

## 4. Dopaminergic modulation of long-term synaptic plasticity in mPFC

Via the mesocortical dopaminergic pathway, mPFC receives direct projections from dopaminergic neurons residing in the ventral tegmental area (VTA) and, to a lesser extent, in the substantia nigra pars compacta (Thierry et al., 1973; Björklund and Dunnett, 2007). An effective dopamine (DA) concentration in mPFC is mainly determined by three processes: release of the neuromodulator by dopaminergic axon terminals, reuptake by DA (DAT) or norepinephrine (NET) transporter membrane proteins in these terminals and metabolism *via* catechol-O-methyltransferase (COMT), an enzyme located in post synaptic neurons or glial cells (Garris and Wightman, 1994; Mundorf et al., 2001; Yavich et al., 2007; Bai et al., 2014). In wild-type mice, pharmacological DAT inhibition had a negligible effect on extracellular prefrontal DA levels, whereas NAT inhibition increased DA levels 2- to 4-fold (Käenmäki et al., 2010), suggesting that DA uptake in this cortex depends primarily on the NET, in contrast to the striatum where it primarily mediated by DAT. In NET- and COMT-knockout mice, prefrontal DA levels were increased by 55 and 60%, respectively (Morón et al., 2002; Käenmäki et al., 2010). The data from DAT-knockout mice are controversial as either no effect (Shen et al., 2004) or a 3.6-fold increase of DA levels (Xu et al., 2009) were observed.

The release profile of DA is determined by the activity of dopaminergic neurons, thought to occur *in vivo* in two distinct firing regimes: “tonic” and “phasic” (Floresco et al., 2003; Grace et al., 2007). The tonic regime corresponds to a regular spontaneous activity of a subset of dopaminergic neurons and is thought to provide a stable baseline DA concentration in target structures, including mPFC. The phasic regime corresponds to burst firing of these neurons, providing a neuronal basis for reward-based learning in the brain (Hollerman and Schultz, 1998). It has been argued that due to differences between striatal and cortical dopaminergic projection systems, the latter is more suited to detect relatively slow (i.e., on the time scale of seconds to minutes) changes in tonic DA concentration and is not sensitive to short (i.e., about 200 ms) phasic dopaminergic signals (Seamans and Yang, 2004; Lavin et al., 2005). This distinction is supported by the data showing that mPFC expresses much slower clearance rates for DA (Garris and Wightman, 1994), leading to a prolonged DA response to short activation bursts of DA neurons. Moreover, prefrontal DA appears to be released by a specific subpopulation of VTA dopaminergic neurons that are molecularly and functionally distinct from “conventional” neurons thought to signal reward-related activity, at least in mice (Lammel et al., 2008). This subpopulation is characterized by the ability to sustain tonic firing at high rates for prolonged periods of time, and by a lack of D2 autoreceptors that inhibit DA release by feedback control. Finally, synaptic plasticity experiments have repeatedly shown that long-term (in the range of tens of minutes), but not short-term, changes in background DA concentration determine the amplitude and direction of synaptic plasticity in the mPFC (Matsuda et al., 2006; Kolomiets et al., 2009). This is in contrast to the striatum, where a short DA pulse concurrently with afferent stimulation can induce a change in the direction of plasticity (Wickens et al., 1996).

On the post-synaptic side, there are two main dopaminergic receptor types, D1 (including D1 and D5 receptors) and D2 (including D2, D3, and D4 receptors), both of which are expressed in prefrontal neurons (Vincent et al., 1993; Gaspar et al., 1995; Santana et al., 2009; Zhang et al., 2010) and are important for normal PFC-dependent behaviors (Seamans et al., 1998; Setlow and McGaugh, 2000; Floresco et al., 2006; St Onge et al., 2011). Santana et al. (2009) performed a quantitative analysis of D1- and D2- receptor expression in the rat prelimbic mPFC, showing that these receptors are present in excitatory pyramidal neurons and, to a lesser extent, in inhibitory γ-Aminobutyric acid (GABA) interneurons in all layers. Much larger proportion of pyramidal neurons express DA receptors in deeper (V-VI) than in superficial (II-III) layers. As to the receptor types, a similar proportion of pyramidal cells expressed D1 and D2 receptors in layer V, while in other layers D1-expressing cells were more abundant (3- to 4-fold) than D2-expressing ones. In mice, D1 receptors are more abundant in deeper layers, while D2 receptors in superficial ones, with D1:D2 ratio varying between 1.5 and 2.5 depending on age (Wei et al., 2018; Cullity et al., 2019; Bjerke et al., 2022). Co-localization studies show that only about 25% of prelimbic cells express both receptor types in rats and mice, suggesting a partial segregation of D1- and D2-expressing neurons in the mPFC (Gaspar et al., 1995; Vincent et al., 1995; Wei et al., 2018). Interestingly however, in several electrophysiological studies the effects of pharmacological manipulations of both D1 and D2 receptors were observed in single pyramidal mPFC neurons (e.g., Tseng and O'Donnell, 2004; Matsuda et al., 2006; Xu et al., 2009). In these studies GABAergic transmission was blocked, excluding the possibility that D2 receptors acted on neuronal activity *via* interneurons (Xu and Yao, 2010).

D1 and D2 receptors are coupled to G proteins stimulating (Gα_s/olf_) and inhibiting (Gα_i/o_) second messenger cAMP, respectively (Missale et al., 1998; Beaulieu and Gainetdinov, 2011). Consequently, *via* their main transduction cascades these receptors exert opposite effects on the classical cAMP - PKA plasticity pathway (Malenka and Bear, 2004). Specifically to the PFC, the D1-mediated facilitatory effect on LTP can be mediated by such mechanisms as stimulation of surface expression of AMPA receptors (Sun et al., 2005), neuronal excitability increase *via* protein kinase C-phospholipase C cascade (Tseng and O'Donnell, 2004; Chen et al., 2007), and potentiation of NMDA receptor responses (Zheng et al., 1999; Li et al., 2009). The D1-mediated activation of cAMP-PKA pathway has been shown to be necessary for LTP in hippocampal (Jay et al., 1998; Gurden et al., 1999) and callosal (Coppa-Hopman et al., 2009) mPFC synapses *in vivo*. Some of these D1-mediated actions are mirrored by antagonistic D2-activated cellular pathways. In particular, D2 receptors decrease excitability, downregulate AMPA trafficking, and suppress NMDA receptor activity (Zheng et al., 1999; Wang et al., 2003; Tseng and O'Donnell, 2004; Sun et al., 2005). In addition, D2 receptors modulate redistribution of NMDA receptors away from the synapse and inhibit CaMKII, preventing LTP (Xu et al., 2009). Cooperative effects of these two receptor classes have also been reported. For example, coactivation of D1/D2 receptors leads to an increase in intracellular calcium levels in cell cultures (Lee et al., 2004) and to the activation of the MAPK/ERK, required for both LTD and LTP in *in vitro* (Otani et al., 1999; Kolomiets et al., 2009).

A given DA concentration will thus activate both D1 and D2 receptors in the local circuit, such that their dynamic balance will determine the type of resulting plasticity (or its absence), depending on differential sensitivities of downstream molecular cascades to DA (Nomura et al., 2014). A certain controversy exists concerning the relative activation of prefrontal D1 and D2 receptors as a function of prefrontal DA levels. A widely held hypothesis is that lower DA concentrations predominantly activate D2 receptors, while higher DA concentrations result in a stronger net D1 activity (Goto and Grace, 2005; Shen et al., 2008). This hypothesis is based on earlier studies suggesting that a larger proportion of D2 receptors are in a high affinity state, and, conversely, a larger proportion of D1 receptors are in low affinity state in the rat striatum (Creese et al., 1983; Richfield et al., 1989). However, this issue is far from clear in relation to PFC. In fact, prefrontal studies have repeatedly reported D1-mediated effects at a lower DA concentrations and D2-mediated effects at a higher one (Zheng et al., 1999; Trantham-Davidson et al., 2004; Li et al., 2009), contrary to the striatum-based data. This can occur due to several reasons: (i) the density of D1 receptor distribution in rodent PFC is higher than that of D2 receptors (see above); (ii) differences in the localization of DA receptors (synaptic for D2 vs. extra-synaptic for D1) may contribute to receptor sensitivities to DA (Seamans and Yang, 2004); (iii) the distribution of affinity states of these receptors may differ between the striatum and PFC (no studies, to our knowledge directly addressed this question so far). Affinity states may change receptor sensitivities to DA by several orders of magnitude (Richfield et al., 1989).

While in primates the study of the direct influence of DA on long-term memory and synaptic plasticity is hard and only correlative evidence exists (see Puig et al., 2014, for review), experiments in rodents have clearly demonstrated that this neuromodulator is strongly involved in executive behavioral control (Floresco et al., 2006), while also strongly influencing the amplitude and direction of prefrontal synaptic plasticity (Jay, 2003; Otani et al., 2003). Elucidating the role of DA in plasticity may thus help to understand its role in mPFC-dependent memory and therefore in executive behavior. However, it has proved difficult to provide a coherent interpretation of results of synaptic plasticity experiments, since it is complicated by the fact that DA effects on plasticity depend on the stimulation length and frequency, as well as on the length and time of application. One of the objectives of this review is to provide such a unifying interpretation by means of plasticity curves introduced in the previous section. The next sections provide an overview of experimental studies, including those from our group, that address DA modulation of prefrontal synaptic plasticity in rats and mice (see Table 2).

**Table 2:** Experimental studies on DA modulation of long-term synaptic plasticity in rodent PFC.

**Rats**				
	**Type**	**Freq. (Hz)**	**Mol. Casc**.	**References**
*In vivo*	LTP	250	+D1, +NMDA, +PKA, −D2	Gurden et al., 2000
*In vitro*	LTP	300	+D1, +NMDA, +PKA, +prot. synth	Huang et al., 2004
	LTD/LTP	50–100	+NMDA	Law-Tho et al., 1995
		50	+D1, +D2, +mGluR, +MAPK	Otani et al., 1999
		50	+NMDA, +mGluR, +PLC, +PKC, +IP3	Otani et al., 2002
		50	+D1, +D2, +NMDA, +ERK	Kolomiets et al., 2009
	LTD	3	+D1, +D2, +NMDA, +PLC, +PKC, +MAPK	Bai et al., 2014
**Mice**				
*In vitro*	LTP	300	+D1, +prot.synth	Huang et al., 2004
		100 × 5		Xu et al., 2009
		100	+NMDA	Cui et al., 2011
	LTD	3	+D1, +D2, +PKA, +mGluR, -NMDA	Huang et al., 2004
		10	+eCB, +mGluR, +PLC, −NMDA, −D1, −D2	Lafourcade et al., 2007

In all experiments, a tetanic stimulation at the given frequency was applied, except the study by Xu et al. (2009) where a theta-burst stimulation was used (five 100 Hz bursts with 200 ms intervals). Plus (minus) signs in the 4th column indicate that the corresponding receptor/enzyme was (was not) required for a given type of plasticity.

### 4.1. Dopaminergic modulation of synaptic plasticity in the rat

The only *in vivo* data on DA modulation of prefrontal plasticity comes from studies in anesthetized rats, in which an electric stimulation of VTA dopaminergic afferents to mPFC, concurrent with tetanic plasticity induction protocol at 250 Hz, enhanced NMDA-dependent LTP in hippocampal-medial prefrontal synapses (Gurden et al., 1999). An electrolytic lesion of VTA impaired this LTP, while saturation of LTP by stimulation at a higher frequency (300 Hz) eliminated the DA-induced enhancement effect, suggesting that DA acts on plasticity in a limited window of stimulation frequencies. The enhancement of LTP was mediated by D1 receptor activation *via* cAMP-PKA cascade, while manipulation of D2-receptor activation by agonists or antagonists had no effect (Gurden et al., 2000).

*In vitro*, LTP induced by 300 Hz stimulation was strongly reduced by D1 receptor antagonism or PKA inhibition, while D2 receptor blockade had no influence (Huang et al., 2004), in a good agreement with the *in vivo* data above. In the range of lower stimulation frequencies (50–100 Hz), a short and strong DA application (100 μM for 5 min during stimulation) shut down LTP and favored NMDA-independent LTD (Law-Tho et al., 1995; Otani et al., 1998). This LTD required combined activity of D1, D2 receptors and mGluRs, and also involved MAP kinases (Otani et al., 1998, 1999). In a stark contrast to the LTD facilitation by a short-term high-concentration DA bath, a long-term application of low-concentration DA (3 μM for 15–40 min) resulted in an opposite effect, namely LTP facilitation and conversion from LTD to LTP (Matsuda et al., 2006; Kolomiets et al., 2009). Moreover, in the LTP regime, the amplitude of potentiation was highest for an optimal DA concentration, such that too low or too high concentration of DA abolished LTP (Kolomiets et al., 2009). Molecular mechanisms implicated in the DA-facilitated LTP include co-activation of D1, D2 receptors and MAP kinases, similarly to DA-induced LTD above, but also require NMDA receptor activity.

Out of the two different forms of LTD induced by low-frequency stimulation *in vitro*, NMDA-dependent (Bai et al., 2014) and NMDA-independent (Caruana et al., 2011), only the former was tested in different DA conditions. In that study we have shown that the NMDA-dependent LTD required a co-activation of D1 and D2 receptors, since the antagonism of either receptor blocked plasticity (Bai et al., 2014). Moreover, when endogenous DA activity was augmented by DAT inhibition during stimulation, the LTD was blocked by overstimulation of D1 receptors. Since in these experiments too weak or too strong DA receptor activation impaired LTD, these results can be interpreted in terms of an inverted-U shape profile, as has been done for LTP results above (Bai et al., 2014; Otani et al., 2015). Molecular mechanisms underlying this NMDA-dependent form of LTD involved PLC, PKC and MAP kinases.

With the help of the plasticity curves introduced earlier, DA modulation of plasticity in rats can be interpreted in the following way (see Figure 2B). First, in the regime of high stimulation frequencies (≥200 Hz), in which only LTP can be induced, DA modulates this LTP *via* D1 receptors acting through cAMP-PKA cascade. Available evidence suggests that D2 receptors are not involved in the LTP induced at these high frequencies, neither *in vivo* nor *in vitro*. Second, near plasticity threshold (around 50 Hz *in vitro*), DA *via* both D1 and D2 receptors exerts strong bidirectional effect on plasticity. In particular, high DA elevation induces a rightward shift of the LTD/LTP threshold toward higher synaptic activities (high DA, conversion from LTP to LTD, Figure 2B), while low DA elevation induces a leftward shift (low DA, conversion from LTD to LTP). It follows that progressive elevation of DA concentration near threshold should peak at some optimal DA concentration exhibiting the “inverted-U” LTP profile, in agreement with the data (Kolomiets et al., 2009). Finally, still lower stimulation frequencies enter into LTD-only regime, in which DA controls LTD in a bidirectional manner: either DA decrease or DA elevation can shut down LTD *via* cooperative action on D1 and D2 receptors to affect plasticity. In this regime, the interpretation of experimental results within the proposed framework is complicated by our ignorance about the stimulation-induced synaptic state relative to the LTD/LTP threshold. For example, if the synaptic state is far from the threshold (i.e., close to the baseline synaptic activity), then progressive DA elevation will shut down LTD (as in the study by Bai et al., 2014). On the other hand, if the synaptic state is close to the LTD/LTP threshold, then DA elevation will enhance LTD (as is the case with mouse LTD, Huang et al., 2004, see below). Thus, our hypothesis of DA modulation is neither supported nor disproved by these opposite experimental results on LTD. However, the hypothesis predicts that for a given synaptic state, progressive elevation of DA concentration will result in a particular response profile of synaptic efficacy change, depending on how close the synaptic state is to the plasticity threshold.

It can further be speculated that the same pattern of DA plasticity modulation occurs *in vivo*, since the same molecular mechanisms are likely to be at play. However, in this condition the strongest dopaminergic modulation of plasticity is predicted to occur at much lower stimulation frequencies (corresponding to a lower LTD/LTP threshold *in vivo*) and in a narrower frequency window (see Figure 2A), a prediction that is yet to be tested.

### 4.2. Dopaminergic modulation of synaptic plasticity in the mouse

While *in vivo* mouse data testing DA modulation of prefrontal plasticity is currently absent (to our knowledge), *in vitro*, D1 agonists facilitated LTP induced at 300 Hz (Huang et al., 2004), in agreement with the role of D1 receptors in this form of LTP in rats (without agonist application, a stable but weak LTP was observed in this study).

A synaptic stimulation at an intermediate strength, in particular, a tetanic stimulation (at 100 Hz) or a theta-burst stimulation, induced LTP in wild-type mouse slices (Xu et al., 2009; Xu and Yao, 2010). This LTP could not be induced in slices from DAT-knockout mice by these same protocols. This LTP impairment in the mutant mice has been shown to result from DAT-induced elevated tonic DA concentration, which acted *via* D2 receptors to activate protein phosphatase 1 and block LTP. This effect was confirmed in experiments with wild-type mice, since when these mice were injected amphetamine or DAT inhibitor (both of which induce elevated DA levels in the mPFC) 30 min before killing and slice preparation, LTP was blocked also in these mPFC slices (Xu et al., 2009). Thus, in mice, as in rats, an elevated tonic DA concentration shuts down LTP, induced at frequencies around 50–100 Hz, although the underlying molecular mechanisms could be different in the two species. In the framework of the proposed hypothesis, the LTP block is explained by a DA-induced shift of the plasticity threshold toward higher synaptic activities (see Figure 2B).

In the low-frequency regime, LTD induced at 3 Hz was blocked by antagonists of D1 and D2 receptors or by PKA inhibition, but enhanced by a high-concentration DA bath *via* D1 receptor activation (Huang et al., 2004). As discussed earlier, this might be an indication that the stimulation-induced synaptic state in this study was closer to the LTD/LTP threshold, rather than to the baseline synaptic state. Contrary to the observed DA modulation of the LTD at 3 Hz, blockade of DA receptors did not affect LTD induced at 10 Hz (Lafourcade et al., 2007). If confirmed by further research, these data would support the existence of DA-dependent and DA-independent forms of low-frequency induced LTD in mice, as in rats.

### 4.3. STDP experiments

In contrast to classic LTD/LTP induction protocols considered earlier, spike-timing dependent plasticity (STDP) protocols induce synaptic changes by pairing a pre-synaptic stimulation (usually an extracellular current pulse delivered to the layer where synaptic connections of the recorded neuron reside), with a post-synaptic spike evoked by an intracellular current injection. Such a paired stimulation is repeated 50-100 times at a low frequency (about 0.1 Hz). The polarity of plasticity depends on whether the pre-synaptic stimulation comes before (leading to LTP in hippocampal cultured neurons and neocortical slices) or after (leading to LTD) the post-synaptic spike (Markram et al., 1997; Bi and Poo, 1998). In STDP experiments targeting mPFC, pyramidal neurons in layers V are usually recorded, with the presynaptic stimulation applied to layer II-III. In what follows, the timing difference Δ*t* between the presynaptic stimulation and the post-synaptic spike is denoted as positive for pre-post pairing, and negative for post-pre pairing. To distinguish between the classical LTD/LTP and that induced by the STDP protocol, we will denote the latter “t-LTD/t-LTP.”

A number of studies applied STDP protocols to study prefrontal long-term plasticity in rats and mice, including its modulation by DA, as well as by other neuromodulators (Couey et al., 2007; Xu and Yao, 2010; Goriounova and Mansvelder, 2012; Zaitsev and Anwyl, 2012; Ruan et al., 2014; Louth et al., 2021). These experiments have shown that regulation of plasticity by pre-post timings in mPFC is different from that in the classical experiments, since in the absence of exogenous DA and with blocked inhibitory (GABA) receptors, LTP was induced by pre-post stimulation at Δ*t* = +5, +10 ms, whereas no plasticity could be induced either by Δ*t* = +30 ms or Δ*t* = −30 ms. An application of a high DA concentration (20–100 μM) during pairing extended t-LTP portion of STDP to all the tested time intervals (Xu and Yao, 2010; Ruan et al., 2014). This DA-facilitated t-LTP was D1-cAMP-PKA dependent and required NMDA activation. Pharmacological modulation of D2 receptor activity did not affect plasticity directly, but only indirectly *via* inhibition of GABA release by interneurons (Chiu et al., 2010; Xu and Yao, 2010). In the presence of GABA, t-LTD or no change have been observed at almost all tested intervals (in both young adult and mature mice, Louth et al., 2021). Optogenetic activation of VTA DA fibers blocked t-LTD and this modulatory effect was abolished in the presence of the D2 receptor antagonist, but was not affected by the D1 receptor antagonist (Louth et al., 2021). The blockade of t-LTD by D2 receptors is consistent with the evidence of D2-mediated GABA suppression by DA acting by inhibiting its presynaptic release (Xu and Yao, 2010).

A comparison of the STDP data with results from classical induction protocols reviewed earlier suggests that the observed t-LTP corresponds to D1-NMDA-PKA-dependent (and D2-independent) classical high-frequency LTP regime. This suggestion is further supported by the data showing that a high concentration of applied DA in mouse slices was shown to enhance a weak classical LTP (Huang et al., 2004).

### 4.4. Role of other neuromodulators in prefrontal long-term plasticity

Other neuromodulators present in mPFC, such as noradrenalin (NA), acetylcholine, and serotonin, have been reported to act separately or in concert with DA to affect synaptic plasticity.

Dendritic spines of cortical pyramidal neurons appear to be a common target of both DA and NA inputs, and both receptor types share similar signaling cascades, which can modulate excitatory as well as inhibitory synaptic transmission (see Xing et al., 2016, for review). *In vivo*, locus coeruleus stimulation concurrently with a high-frequency stimulation of hippocampal-to-PFC inputs enhanced LTP in rats (Lim et al., 2010), similarly to the enhancement of LTP by VTA stimulation (Gurden et al., 1999). *In vitro*, an elevated concentration of NA induced NMDA-dependent LTD in layer I-II to layer V fibers *via* postsynaptic α-adrenoceptors and molecular cascade involving PKC and MAP kinase (Marzo et al., 2010). In slices from amphetamine-treated mice, in which extracellular levels of monoamines (DA, NA and serotonin) are increased, compared to wild-type controls, a low amphetamine dose enhanced, while a high amphetamine dose abolished, an LTP induced by theta-burst stimulation (Xu and Yao, 2010). This effect is similar to that observed with high and low levels of DA (Otani et al., 1998; Kolomiets et al., 2009). The low-dose LTP facilitation depended on D1 and β-adrenoceptor activation *via* cAMP-PKA cascade. In contrast, the high-dose LTP blockade was mediated by D2 receptors. In layer III rat pyramidal neurons, β2 adrenoceptor agonist increased the amplitude of t-LTP (Δ*t* = +10 ms) *via* both postsynaptic signaling by PKA and presynaptic suppression of GABAergic inhibition (Zhou et al., 2013). This is somewhat reminiscent of t-LTP enhancement by both post- and pre-synaptic mechanisms by DA in mice (Xu and Yao, 2010). These data suggest cooperative action of DA and NA on synaptic plasticity.

Concerning the role of prefrontal acetylcholine receptors, it has been shown that an activation of nicotinic receptors (nAChR) on several interneuron types in mouse mPFC can increase GABAergic signaling and prevent t-LTP (Δ*t* = 5 ms) in pyramidal layer V neurons (Couey et al., 2007; Goriounova and Mansvelder, 2012). On the other hand, an activation of muscarinic acetylcholine receptors (mAChR) in rat pyramidal neurons can induce an activity-independent LTD *via* PLC-PKC cascade (Huang and Hsu, 2010) and convert transient to permanent LTD (induced by low frequency stimulation at about 1 Hz, Caruana et al., 2011). Therefore, endogenous acetylcholine, that stimulates both receptor types, can potentially contribute to LTD induced at low stimulation frequencies around 1 Hz, in both rats and mice.

Lastly, serotonin depletion was shown to result in a LTP enhancement in the hippocampal-prefrontal pathway in rats *in vivo* (Ohashi et al., 2003). Serotonin application *in vitro*, together with tetanic stimulation at 50 Hz, facilitated NMDA-independent LTD *via* mGluR activation and MAP kinase (Zhong et al., 2008). These data suggest that serotonin may also contribute to prefrontal LTD.

In addition to neuromodulatory systems, retrograde signaling was shown to play an important role in prefrontal LTD. Consistent with a general involvement of endocannabinoids in retrograde signaling and cortical LTD (Heifets and Castillo, 2009), they have been implicated in both activity-independent and tetanus-induced LTD in rodent mPFC. In particular, a sole application of cannabinoid agonists and antagonists induced LTD and LTP, respectively, in rat layer V pyramidal neurons (albeit only in a subset of tested cells, Auclair et al., 2000). Consequently, when these pharmacological agents were applied together with high-frequency tetanic stimulation (at 100 Hz), they biased the tetanus-induced plasticity toward LTD or LTP. In mice, a low-frequency stimulation at 10 Hz induced LTD that was completely blocked by endocannabinoid antagonists acting *via* mGluR-PLC cascade (Lafourcade et al., 2007). Cannabinoid receptors (CB1) were also shown to be involved in controlling prefrontal inhibition. In mice, these receptors co-localize with D2 receptors at about 30% of inhibitory synaptic terminals in the mPFC, so that agonists of either receptor suppress inhibitory transmission (Chiu et al., 2010). Moreover, endocannabinoids may not be the only retrograde messengers in the mPFC, as the study by Huang and Hsu (2010) reported the implication of nitric oxide as a retrograde messenger during activity-independent LTD (see Caruana et al., 2011).

Finally, regulation of long-term plasticity by inhibitory GABAergic transmission has been demonstrated in a number of studies (Couey et al., 2007; Huang et al., 2007; Chiu et al., 2010; Xu and Yao, 2010; Louth et al., 2021). Often the same neuromodulator acts in a cooperative manner on both glutamatergic and GABAergic transmission to influence plasticity (Couey et al., 2007; Xu and Yao, 2010). In most of the synaptic plasticity studies reviewed earlier, GABAergic transmission was routinely blocked to separate a direct influence of neuromodulators on inhibition (which is rather complex, see Seamans and Yang, 2004, for review) from its influence on the plasticity mechanisms *per se*.

### 4.5. Summary of synaptic plasticity mechanisms in the mPFC

A general conclusion that can be made concerning the cellular mechanisms of long-term plasticity in the mPFC and its modulation by DA is that distinct plasticity mechanisms are at play depending on the level of stimulation-induced synaptic activity. In particular, the experimental data consistently show, *in vitro* and *in vivo*, in rats and mice, an involvement of D1 and NMDA receptors, as well as PKA and protein synthesis, in LTP induced at high stimulation frequencies (≥200 Hz). This form of LTP corresponds to the common form of hippocampal LTP (Huang and Kandel, 1995; Navakkode et al., 2007).

In contrast, competing molecular cascades for LTD and LTP mediate plasticity at lower stimulation frequencies around 50–100 Hz *in vitro* and near 3–5 Hz *in vivo*. In particular, at these frequencies LTP enters into competition with NMDA-independent LTD. While both types of plasticity near threshold activate MAP kinases, LTP requires NMDA-receptor activation, whereas LTD depends on mGluR activity. This is consistent with the proposal that these two receptors represent “coincidence detectors” for LTP and LTD, respectively (Karmarkar and Buonomano, 2002; Bender et al., 2006). It is likely that the mGluR-dependent form of LTD acts *via* the retrograde endocannabinoid signaling and is expressed presynaptically as in the somatosensory cortex (Auclair et al., 2000; Bender et al., 2006).

At still lower frequencies (in the range of 1–3 Hz) LTD takes over LTP, and, moreover, new forms of LTD, likely different in rats and mice, start to play a role. In rats, one form of LTD depends on NMDA activity and a cooperative action of D1 and D2 receptors (Bai et al., 2014). The second form of LTD requires mGluR and mAchR activation, but does not depend on either postsynaptic calcium or NMDA receptor activity (Huang and Hsu, 2010; Caruana et al., 2011). It is not known whether this latter form of LTD is under influence of DA. In the study of Bai et al. (2014), no residual LTD was observed at 3 Hz when DA receptors were blocked, suggesting that these two forms of LTD may be successively activated at progressively lower synaptic stimulation levels. More research however is required to support this conclusion, as the activity of acetylcholine receptors was not controlled during this study. At these low frequencies in mice, only NMDA-independent forms of LTD were observed so far (Huang et al., 2004; Lafourcade et al., 2007), in contrast to rats. Both forms of LTD require mGluR activation and endocannabinoid signaling, whereas only 3-Hz LTD was DA-dependent (Huang et al., 2004).

The experimental data strongly suggests that plasticity near threshold is regulated in a bidirectional manner by tonic dopamine *via* both D1 and D2 receptor subtypes. Although other neuromodulators, such as NA and serotonin, may also be involved in different aspects of this plasticity (especially in LTD Zhong et al., 2008; Marzo et al., 2010), only DA has been shown to directly influence the direction of plasticity to our knowledge (Matsuda et al., 2006; Kolomiets et al., 2009; Zhang et al., 2009). A more complex scenario can not be however excluded, in which a particular combination of several neuromodulators (including DA acting *via* D1 receptors) favors LTP, while a different combination (including DA acting *via* D2 receptors) favors LTD (Seol et al., 2007). Such an assumption is hypothetical at present as there are not enough data to support it. In mice, the available evidence suggests that an elevation of tonic DA can shut down LTP induced at 100 Hz *via* D2 receptors (Xu et al., 2009). While there is no direct evidence that tonic DA can change the direction of plasticity in mice, it was shown that DA facilitates LTP induced at higher stimulation frequencies, while it facilitates LTD at low frequencies, as discussed earlier. Therefore, it is reasonable to suggest that DA effect on plasticity switches sign at some intermediate stimulation strength (likely in the range of stimulation frequencies between 10 and 100 Hz *in vitro*).

## 5. Computational models of prefrontal long-term memory and its modulation by dopamine

The processing vs. representational dichotomy adopted by Wood and Grafman (2003) to classify high-level models of PFC function can also be applied to describe a large variety of computational models of this structure in the way they rely on long-term memory storage. Processing-type models primarily focus on the ability of PFC neurons to show elevated persistent activity in delay-period tasks, considered to be a neural implementation of working memory (Durstewitz et al., 2000; O'Reilly, 2006). A wide array of such models differ in neuronal mechanisms that are proposed to support persistent activity, but they all share the property that such activity can maintain in the working memory *any* information relevant for the task. The unique role of PFC is therefore characterized by the operation it can perform, rather than by the nature of the stimuli it operates with. In simple working memory tasks, the persistent network state may represent the direction of eye movement to a remembered visual cue (Compte et al., 2000), whereas it can represent a high-level contextual cue in a hierarchical goal structure or an abstract behavioral rule (Rougier et al., 2005; O'Reilly and Frank, 2006). In these models, the role of long-term plasticity is often implicit and is constrained by the requirement to ensure persistent activity states. For example, an item can be held in working memory by recurrent excitation of neurons interconnected with high synaptic weights, forming a point attractor (Durstewitz et al., 2000). In this case it is assumed that PFC learns slowly over time all the items it can represent, such that the corresponding attractor state could be activated in working memory when needed (Amit and Brunel, 1997). Alternatively, a synaptic matrix can be constructed so as to enable continuous attractor dynamics, in which case the attractor represents the location of a remembered object rather than the objects itself (Camperi and Wang, 1998).

The role of dopamine in processing-type models has been modeled in relation to working, but not long-term, memory. In particular, in a detailed biophysical model (Durstewitz et al., 2010) proposed that an elevated DA by acting primarily on D1 receptors, enhanced the stability of persistent states and their resistance to distractors *via* combined action on GABA and NMDA currents. This state corresponds to active maintenance of important information in working memory, e.g., during a behavioral task. In low DA conditions, D2 receptors dominate and produce opposing destabilizing effects, allowing the contents of working memory to be rapidly updated (Dreher and Burnod, 2002; Seamans and Yang, 2004). This model, together with an earlier model of gain modulation by DA (Servan-Schreiber et al., 1990), made sense of a multitude of DA effects on neural activity and provided a solid biophysical foundation to more abstract models of working memory (O'Reilly, 2006).

In contrast to processing-type models above, representational models can be distinguished by the type of information that is proposed to be stored by PFC. Models of human cognition focused on behavioral planning (Dehaene and Changeux, 2000), learning hierarchical behavioral structures (Cooper and Shallice, 2006; Botvinick, 2008; Holroyd and Mcclure, 2015) and associations between stimuli, rewards, actions and their outcomes (Alexander and Brown, 2011; Soltani and Koechlin, 2022). Computational models of rodent behavior focused on strategy selection (Dollé et al., 2010; Sheynikhovich and Arleo, 2010), goal-directed behavior and action planning (Hasselmo, 2005; Martinet et al., 2011). In the latter models PFC neurons learned either a representation of a behavioral strategy (e.g., response- vs. place-based) or the structure of a goal-directed task. These high-level models are not specific about neuronal mechanisms supporting information storage and usually assume a combination of associative Hebbian and reinforcement learning rules. The role of DA is either not considered at all or is assumed to signal reward-prediction error in the context of standard reinforcement learning algorithms. Given the wealth of evidence, reviewed earlier, that DA is a powerful modulator or long-term synaptic plasticity in mPFC, we attempted to unify available data in a simple computational model, in which DA shifts the position of the LTD/LTP plasticity threshold and therefore controls the induction of plasticity (Sheynikhovich et al., 2011, 2013).

**Figure 3:**
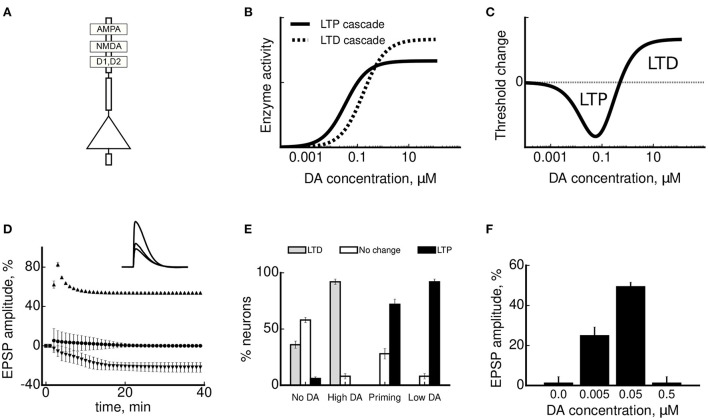
A model of DA-modulated synaptic plasticity and simulation results. **(A)** A schematic representation of a multicompartment model of pyramidal neurons with AMPA, NMDA, D1 and D2 receptors. **(B)** A molecular cascade for LTP is facilitated at lower DA concentrations by D1 receptors (shown by the full black line). A molecular cascade for LTD is facilitated at higher DA concentrations by D2 receptors (dashed line). **(C)** The plasticity threshold between LTD and LTP shifts as a function of a difference between the activities of the two molecular cascades in **(B)**, resulting in a U-shaped net effect of DA concentration on synaptic plasticity. A negative threshold change corresponds to its shift to lower stimulation frequencies in Figure 2B and facilitation of LTP. Conversely, a positive change facilitates LTD. **(D)** Synaptic plasticity in the model neurons (measured by the amplitude of somatic EPSP±SD) as a result of tetanic stimulation at low (3 Hz, shown by downward triangles), intermediate (50 Hz, circles) and high (300 Hz, upward triangles) stimulation frequencies. Inset: mean EPSP amplitude 40 min after stimulation in the three cases. **(E)** Dopaminergic modulation of synaptic plasticity near LTD/LTP threshold. The bars show the proportion of simulated neurons that underwent LTD (in gray), no change (in white) or LTP (in black), depending on DA conditions. **(F)** An inverted-U-shape dependence of the LTP amplitude for different concentrations of DA, as observed experimentally by Kolomiets et al. (2009). The plots are adapted with permission from Sheynikhovich et al. (2013).

The model extends the classical calcium-based plasticity rule (Lisman, 1989; Shouval et al., 2002) by implementing the threshold modulation *via* opponent activation of D1- and D2-receptor pathways. In a first, calcium-dependent component of the model, the amplitude of synaptic efficacy change in the model neuron (Figure 3A) follows a BCM-like dependence, meaning that *(i)* calcium elevation is necessary for any plasticity to take place; *(ii)* moderate calcium levels result in a decrease of synaptic strength; *(iii)* high calcium levels result in an increase of synaptic strength; and *(iv)* there is a threshold calcium concentration at which LTD is converted to LTP. The postsynaptic calcium concentration in the model is controlled by the calcium influx *via* NMDA channels and *via* high-voltage-activated calcium channels, as observed in real mPFC neurons (Seamans et al., 1997). The second, DA-dependent, component of the model describes how an extracellular tonic DA concentration can modulate the calcium-based plasticity rule. In agreement with the analysis of the *in vitro* experimental data (see Figure 2B), an activation of a D1-mediated molecular cascade facilitates LTP and shifts the threshold to lower calcium concentrations, whereas that of a D2-mediated cascade facilitates LTD and induces a shift in the opposite direction. While in this model we assumed that both D1 and D2 receptors are expressed in single pyramidal neurons, it does not exclude the possibility that the two receptor classes act *via* segregated circuits (Xu et al., 2009), as long as they exert opposing effect on plasticity in target neurons. The effective plasticity modulation by DA is then proportional to the difference between the activities of the two molecular cascades. Because of distinct affinities of D1 and D2 receptors in the model (Figure 3B), as in real mPFC neurons (Zheng et al., 1999; Trantham-Davidson et al., 2004; Li et al., 2009), this difference has an inverted-U-shape dependence on DA concentration (Figure 3C). As a result of such modulation, lower DA concentrations lead to a leftward shift of LTD/LTP threshold and a net facilitation of LTP (corresponding to the downward deflection in Figure 3C), whereas higher DA concentrations leads to a rightward shift of the threshold and a net facilitation of LTD (corresponding to the upward deflection in Figure 3C). To show the effect of various DA conditions on synaptic plasticity, many neurons with slightly different neuronal parameters (i.e., compartment sizes and properties of ionic currents) were simulated to reflect differences between real neurons in a slice. These neurons were then tested under conditions mimicking *in vitro* experimental protocols reviewed in Section 4. Modeling results show that for stimulations at high (300 Hz), intermediate (50 Hz, near LTD/LTDP threshold) and low (3 Hz) frequencies all model neurons exhibited LTP, no change, or LTD, respectively (Figure 3D), similarly to standard plasticity models (Shouval et al., 2002; Clopath et al., 2008). At the high and low frequencies, changes in the stimulation protocols (stimulating at the same frequency for a longer or shorter period of time) could only saturate or abolish plasticity (not shown). However, near threshold, where standard protocols did not induce any plasticity, increasing the stimulation length produced either LTP, LTD or no change in different neurons (“No DA” condition in Figure 3E). We hypothesize that this variability in the induced plasticity in the model neurons reflects the variability of synaptic plasticity near this frequency in real neurons *in vitro*, observed experimentally (Hirsch and Crepel, 1990; Law-Tho et al., 1995; Otani et al., 1998; Kolomiets et al., 2009).

In agreement with the key results of plasticity experiments in the mPFC, DA strongly influenced both the sign and amplitude of plasticity near threshold in the model. In particular, LTD was facilitated by a short-term high-concentration DA application in most of the simulated neurons, as in real neurons (Law-Tho et al., 1995; Otani et al., 1998), because this high DA concentration shifted the threshold to higher calcium concentrations on the timescale of several minutes (“High DA” condition in Figure 3E). In contrast, LTP was facilitated by a prolonged low-concentration DA bath (Kolomiets et al., 2009), because lower concentrations induced a slow (on the time scales of tens of minutes) shift of the plasticity threshold to lower calcium levels (“Low DA” condition in Figure 3E). Third, the same model provides an explanation of DA priming effect, showing that a short DA application 40 min before stimulation changes the direction of plasticity in mPFC neurons (“Priming” condition in Figure 3E, Matsuda et al., 2006). This is explained by the adaptive threshold dynamics as a function of DA concentration during washout: initially high DA concentration facilitated LTD, while at a later time the concentration decreased and entered the LTP facilitation regime. Finally, the model is also consistent with the experimentally observed inverted-U dependence of LTP amplitude as a function of DA concentration (Figure 3F; Kolomiets et al., 2009). This form of dependence in the model is a direct consequence of the U-shaped dependence of the threshold position on DA concentration (see Figure 3C).

While the model described above is the only one, to our knowledge, addressing the role of DA in prefrontal plasticity, many previous models considered its role in learning in other brain areas, such as the striatum and the hippocampus. It is therefore of interest to compare the key properties of these models.

On a single neuron level, the role of phasic DA signals in reward-based plasticity, thought to occur in the striatum, has been addressed in a number of phenomenological models of reward-based STDP (reviewed in Frémaux and Gerstner, 2015). In these models, synaptic weight change is proportional to the product of the phasic DA signal (relative to baseline) and a Hebbian STDP term, consisting of a potentiation part (for pre-post pairs of spikes) and a depression part (for post-pre pairs). Thus, a positive DA deflection from baseline during a short period of phasic activity should facilitate plasticity, while a negative DA deflection should inverse the sign of plasticity at these synapses. Both of these effects are in principle compatible with what have been observed in experiments in striatal slices, although the sign flipping effect is controversial (Fino et al., 2005; Pawlak and Kerr, 2008; Shen et al., 2008). A common underlying assumption in reward-based models is that a phasic reward signal arriving some time after activation of a synapse (induced by execution of an action) gates permanent changes at this particular synapse. A memory trace of the activated synapses is proposed to reside in a synapse-specific “eligibility trace” or a “synaptic tag” (Frey and Morris, 1997; Gerstner et al., 2018). These properties are not compatible with prefrontal plasticity model described above, mainly because the timescale of DA action on prefrontal plasticity does not suit stringent timing requirements of reward-based learning (Seamans and Yang, 2004). Experimentally, both positive events, such as novelty or food delivery, and negative events, such as stress, increase mPFC DA levels for prolonged periods (Yoshioka et al., 1996; Lavin et al., 2005) and a prolonged application of low DA concentration (15–40 min) is required to switch the sign of plasticity in the model and experiments (Kolomiets et al., 2009). In agreement with these observations, a disruption of phasic firing of DA neurons (leaving tonic firing intact) did not impair performance in a mPFC-dependent working memory task, but did impair acquisition of reward-depended behaviors attributed to the striatum (Zweifel et al., 2009). Moreover, phasic optogenetic phasic stimulation of VTA-PFC fibers time-locked to the reward delivery failed to improve performance in a place preference task (Ellwood et al., 2017).

In the hippocampus, the role of DA in plasticity differs in three principal ways from that in the striatum. First, a pharmacological block of phasic DA activity (leaving tonic DA firing intact), critically important for striatal learning, does not impair long-term spatial memory, nor novelty-induced exploration, two tasks that are thought to depend on the hippocampal DA (Zweifel et al., 2009). Second, presentation of a novel stimulus that preceded (or followed) reward learning by tens of minutes, improved hippocampal long-term memory *via* DA-dependent pathway (Wang et al., 2010). This is in contrast to striatal reward-based learning paradigms, in which correct stimulus-actions associations are stamped-in by a brief phasic elevation of DA after, but not before, action learning. Third, STDP experiments in brain slices and cultured hippocampal neurons have shown that DA expands the effective time window for synaptic potentiation to negative pre-post pairings *via* the activation of D1 receptors and shuts down t-LTD (Zhang et al., 2009; Brzosko et al., 2015). This is in contrast to striatum, where D1 was shown to be necessary for both t-LTP (for positive pre-post timings) and t-LTD (for negative ones, Pawlak and Kerr, 2008). Synaptic tagging and capture hypothesis (Frey and Morris, 1997), positing that DA is involved in the synthesis of plasticity-related proteins, accounted for much of these data, together with earlier evidence that DA is important only for late but not early LTP (Huang and Kandel, 1995; Navakkode et al., 2007; Lisman et al., 2011). It has also inspired computational models of spike-based learning (in either single-neuron setting or in populations of neurons) that successfully reproduced a large array of phenomena observed in electrophysiological and behavioral experiments (Clopath et al., 2008; Ziegler et al., 2015; Gerstner et al., 2018). In these models, the amount of DA directly facilitates synthesis of plasticity-related proteins, which in turn gates the entry of a synapse into a stable state corresponding to late LTP or LTD. Plasticity-related proteins in these models act to switch off a putative “write protection” mechanism, shared by both types of plasticity. This results in a stabilization of transient synaptic changes induced by a weak stimulation, in the presence of novelty-induced elevation of DA. In these models, the amplitude of plasticity (but not its sign) depends on DA, as changes in DA levels control the maintenance, but not the induction, of plasticity, in contrast to the reward-based learning in the striatum.

Given the comparable time scale of DA influence on plasticity in PFC and the hippocampus as well as their apparent insensitivity to phasic DA inputs, the question arises whether the experimental data from mPFC can be accounted for by the synaptic tagging and capture hypothesis of hippocampal learning and related computational models. As mentioned above, in these models DA “stamps in” the state of a synapse that has been tagged for LTD or LTP, whereas the induction process is DA-independent (Wise, 2004; Lisman et al., 2011; Frémaux and Gerstner, 2015). Therefore, higher or lower DA concentrations lead to a higher of lower number of consolidated synapses. It is not clear then, how such a mechanism could explain the bidirectional control of synaptic plasticity, induced by an increase in DA concentration, as has been observed in rodent mPFC (Matsuda et al., 2006; Kolomiets et al., 2009; Xu et al., 2009; Bai et al., 2010).

## 6. Conclusions and perspectives

The experimental data and theoretical models reviewed above support the conclusion that rodent mPFC is directly involved in a behaviorally important long-term memory storage, that synaptic machinery in this structure is well adapted to support such storage, and that the properties of dopaminergic modulation of these plasticity mechanisms are rather unique. Electrophysiological studies suggest that this modulation is strongest within biologically plausible stimulation regimes and DA concentrations. At the same time behavioral studies demonstrate dopaminergic involvement in the same experimental paradigms in which plasticity was tested (Pezze and Feldon, 2004; Floresco et al., 2006; Hitchcott et al., 2007). Less data are available concerning the influence of other neuromodulators on plasticity. At present, it appears that NA shares many properties with DA in terms of its influence on plasticity, due to common intracellular signaling pathways (Xing et al., 2016), although direct evidence for the bidirectional influence on plasticity is scarce.

We argued that the available data on dopaminergic influence in prefrontal plasticity is consistent with the idea that tonic DA levels determine the position of the threshold between LTD and LTP, on the time scale from minutes to tens of minutes. This proposed role of DA is distinct from, but not contradictory to, the other hypotheses of dopaminergic function, namely, DA enabling the late phase of plasticity (in the hippocampus, Lisman et al., 2011) and as a reward signal for stimulus-response learning and strategy selection (in the striatum, Goto and Grace, 2005; Collins and Frank, 2014). First, the proposed DA role in the control of the prefrontal plasticity threshold is functionally independent from its role in controlling plasticity-related protein synthesis and late-phase LTP. Moreover, given that in the hippocampus a low DA concentration switches LTD to LTP (Zhang et al., 2009), it is possible that DA controls the plasticity threshold in the hippocampus, as well as in mPFC. One of possible predictions from this hypothesis is that for a correctly chosen plasticity induction protocol (within either classical or STDP frameworks), the switch in the sign of synaptic plasticity following DA application could be observed in the hippocampus. If true, this would extend the functional role of DA in this area compared to hippocampal models. Second, although reward-based coding in the mPFC can not be completely excluded (especially in its anterior cingulate subregion, Holroyd and Mcclure, 2015), available data suggest that, at least in the prelimbic cortex, properties of DA kinetics and its documented influence on plasticity do not suit well for striatum-like reward-based learning paradigms. It could nevertheless be speculated that short phasic DA events, riding on top of slow elevations of DA during behavioral task, transmit reward-based information that could be used for reward-based learning (Frémaux and Gerstner, 2015). Alternatively, it has been argued that DA neurons could transmit reward prediction errors by phasic co-release of glutamate, in parallel to DA (Lavin et al., 2005). Third, it is possible that slow changes in tonic DA, that have a strong influence on prefrontal plasticity, may switch neuronal learning pathways in the mPFC, as in the dorsal (Shen et al., 2008) and ventral (Goto and Grace, 2005) striatum, although at a much slower time scale. However, what these pathways are and whether they are anatomical or functional, is not clear. Anatomically distinct neuronal populations with different DA sensitivities (as in the dorsal striatal MSNs) have not been observed so far (see Vincent et al., 1993, for such a hypothesis). A more likely situation in our point of view is a functional, activity- or frequency-dependent, separation. It has been shown that neocortical neuronal processing occurs in different frequency bands (Sirota et al., 2008) and DA has been implicated in increasing neuronal coherence in theta band (Benchenane et al., 2010). Based on experimental and computational data reviewed above, we proposed that DA exerts the strongest influence on plasticity near threshold, i.e., in theta-gamma frequency range, depending on previous activity (Kirkwood et al., 1996). DA modulation could then switch learning between neurons synchronized at different frequencies (Fries, 2005).

The reviewed data shows that the direction and amplitude of plasticity in mPFC depends in complex ways on the relative activation of D1 and D2 receptors. More specifically, whereas these receptors facilitate opposite plasticity cascades, at low DA concentrations they cooperatively participate in LTD. There is evidence that some molecular cascades mediating synaptic plasticity are activated by a combined action of the two receptor types (Lee et al., 2004). In agreement with this, a number of studies of behavioral flexibility have shown either cooperative or antagonist effects of these receptors on behavior. In particular, it was shown that blockade of either D1 or D2 receptors impaired switches from one strategy to another in a cross maze, leading to an increased number of perseverative errors (Ragozzino, 2002; Mehta et al., 2004; Floresco et al., 2006). This pattern of results suggests that these receptors cooperatively regulate learning to inhibit a previously learned response. The latter study has also shown that D4 receptor blockade *improved* learning, exerting an effect opposite to the other receptors (Floresco et al., 2006). In fear extinction studies, both D1 and D2 receptor antagonists infused in mPFC prevented extinction learning in adult rats (Hikind and Maroun, 2008; Mueller et al., 2010), and D2 agonist quinpirole improved long-term extinction in adolescent rats (Zbukvic et al., 2017). In a simple decision making task where animals had to switch lever pressing from one to another following changes in rewards contingency, both D1 and D2 receptor antagonism increased the number of perseverative lever presses (Winter et al., 2009), suggesting a cooperative action of the two receptors. However, in a more complicated task, in which rats were choosing between a small and sure reward vs. a large and risky one, opposite effect of D1 and D2 receptors was observed (St Onge et al., 2011; Jenni et al., 2017). A similar opposite effect was observed in an instrumental tasks that tested a shift from habitual to goal-directed behavior, thought to be controlled by the mPFC (Barker et al., 2013; Nelson and Killcross, 2013). In particular, in rats and mice overtrained to acquire habitual responses (that were therefore insensitive to contingency degradation and goal devaluation procedures, respectively), D1 antagonist or D2 agonist restored goal-directed behavior, whereas D2 antagonists facilitated habitual responding. A first conclusion that may be derived from the above data is that the role of D1 and D2 receptors in behavioral flexibility differs from their role in working memory, where opposite effects of these receptors on performance have never been observed (El-Ghundi et al., 2007). We therefore propose that antagonistic action of the two receptor classes on behavior is a hallmark of the implication of long-term memory in this behavior. A second conclusion is that the antagonism of the two receptor classes has been mostly observed in tasks where the link between actions and rewards is learned, rather than inhibition of the previously acquired response. The latter can be more dependent on the cooperative action of the two receptor classes, suggesting an important role of LTD.

To conclude, mPFC is critically involved in many cognitive processes, components of executive functions. Most of these processes at some stage rely on long-term memory and synaptic plasticity in PFC neurons, as a large number of experimental studies have demonstrated empirically and most theoretical models have (usually tacitly) assumed. Despite this ubiquitous dependence of executive functions on neuronal mechanisms underlying long-term storage of information, their role is underestimated in current PFC research. It is important to study them, because disturbances in the long-term memory component of executive functions may cause long-term consequences of PFC-dependent mental or age-related disorders. Given a wealth of theoretical models addressing the role of dopaminergic modulation of working, as opposed to long-term, memory, as well as its interactions with striatal control of actions, a challenge for future computational theories is to link these models with prefrontal cortical machinery for storage of long-term memories.

## Author contributions

DS wrote the manuscript with input from SO and JB. AA provided critical feedback and helped shape the research. All authors contributed to the article and approved the submitted version.
